# Structure of Attention and the Logic of Visual Composition

**DOI:** 10.3390/bs4030226

**Published:** 2014-07-30

**Authors:** Katherine Blair Wright, Sonit Bafna

**Affiliations:** School of Architecture, Georgia Institute of Technology, 247 4th Street NW, Atlanta, GA 30030, USA; E-Mail: sonit.bafna@coa.gatech.edu

**Keywords:** visual composition, attention, schema, imaginative function

## Abstract

Two groups of subjects were presented with two façade designs, one with the front façade of the existing Atlanta Public Library, an exercise in modern abstract plastic composition by the Bauhaus-trained architect Marcel Breuer, and the other with alteration that toned down its plasticity and enhanced simple relations of its parts like symmetry and repetition. The subjects were asked to recall and copy the façades. The results showed that while significantly more students recalled elements of the altered façade, the performance was equivocal for the façades for the copying task. However, the copying task showed the subjects making greater errors in reproducing elements and relations on the periphery, and those that reflect a reading of depth in the façades. We present an account of the experiment, making the case that the results show the influence of visual design of the façade on the way that an interested and involved viewer attends to it in the course of parsing and comprehending it. The broader implication of this point is to see the visual design of buildings not as simple means to increase its aesthetic value, but as a sophisticated means to lead the viewer to specific forms of imaginative engagement.

## 1. Introduction

Recent years have seen a re-emergence of interest within the space syntax community on developing systematic procedures for describing formal features of buildings such as visual composition, language, and style—those features that often embody architectural concerns but are not included in descriptions focusing on the organization of space [1,2,3,4]. This paper describes an ongoing set of experiments whose results make specific contributions to this line of research.

It is a common practice in architectural literature, particularly in textbooks on composition and diagramming to describe the quality of composition using terms like symmetry, repetition, contrast, rhythm [5,6,7]. Discovering specific instances of such organizational features in a composition is often understood to be a good way of analyzing a composition and often evaluating it. Few writers have tried to explain why these features should be associated with the quality of composition. The most respected account, so far, seems to be that of Rudolph Arnheim, whose argument uses a language of dynamic organization, of perceived forces between elements of composition, and of opposed expressive qualities like “straightness and flexibility, expansion and contraction, or openness and closeness”, which are both perceived in buildings, and may also be applied to objects and environments at large [5]. Arnheim’s emphasis on our perceptual organization is justified, but his language can now seem quite anachronistic and carries the flavor of just-so explanations—explanations that depend on the very qualities they are called to explain.

We believe that a different type of explanation for the role of such compositional properties in the way the composition functions could be offered. Our explanation depends on the idea of active viewer agency, arguing that compositional properties are not directly a measure of the quality of composition—more symmetry, or more order, does not automatically make a better composition. Rather, what differentiates compositions is the extent to which they allow a viewer (naturally, an appropriately motivated one) to get engrossed in understanding them. What leads the viewer to become engrossed is the activity of discerning different features and relations across the composition, of discovering potential patterns or emergent figures, and of reflecting on any depicted entities that may be present, and so on—an activity that is, therefore, directly influenced by the organizational properties of the composition. A behavioral consequence of this activity should be to distribute the viewer’s attention across the composition, making different parts of it salient.

The proximate aim of our study, then, is first, to test whether the organization of a composition does really produce discernible differences in what is salient within a composition, and second to learn a little more about the mechanism by which this happens. The larger aim is to develop an account of how the buildings take shape in response to a generic functional demand of creating imaginative engagement.

### 1.1. Imaginative Attention and Visual Parsing

We begin by recalling some basic facts about human vision. Human vision is fundamentally inferential; our visual system encounters variation of light intensities on the retina and uses those to develop a useable image of the environment. According to one well established account, the process that is followed first identifies contours, fills them in to create surfaces, and then puts them together to construct individual larger objects as fusions or assemblies of these surfaces [8]. This “parsing” operation relies on visual cues to guide the construction at every step. Because this is an inferential process that operates simultaneously through both bottom-up and top-down processes, parsing makes selective use of the available visual information, using what fits the inferential model and discarding what does not. A pint to note is that much of this process is not in one’s conscious control—as the early gestalt psychologists have observed, we cannot help what we see, nor guide it towards pre-specified ends, although we may control what is in our field of view and use attention to select from within it [9].

This is because we require attention to see [10]; just training our eye on the object of vision is not sufficient. Attention may be essential to our ability to assemble independently observed attributes and features into specific objects and scenes [11]. Attention usually has a focus, which leads to selectively seeing only some aspects of what is in our field of vision. The focus can vary in size and in interest as we choose to either scrutinize something closely using high acuity foveal vision, or to survey an area without any pre-specified goal, maintaining general awareness [9]. Attention itself is task-oriented; to maintain attention on a specific object, we need to be engaged in a visual task concerning the object [12,13]. So in the end, our seeing is task-driven, limited to what we attend to, either purposefully, or as a response to something obtruding into our awareness.

Seeing also has an imaginative dimension. The objects or assemblies constructed may not actually be present, such as when we see a three-dimensional object in marks on a surface or create notional groupings from entities in our field of view. At other times, constructions made can also trigger associations to images in our memory. The importance of this imaginative seeing is to direct thoughts and attention away from what is directly in our field of vision to entities or worlds not immediately present; this is because imaginative seeing can only be sustained by an act of “attentive intentionality” [14].

Given this, we can argue that the manipulation of visual form through composition is not just a simple aesthetic activity—where a designer manipulates the visual form in order to create designs that feel harmonic or pleasing or right [15]—but rather a means to guide the viewer’s attention and, therefore, thoughts in specific directions [16]. This study was designed to test one aspect of this argument: that altering the visual design of a building in particular ways alters a viewer’s attention to it and makes different parts salient.

### 1.2. The Façade as a Case: Marcel Breuer’s Atlanta Public Library

The hypothesis was tested using a façade of a building. The relationship of buildings to their viewers is complex—often designed for viewing by moving visitors, from different angles, and in different environmental conditions. However, in the façade it is possible to find an individual sub-problem in architecture, whose design is mostly driven by visual concerns. We can find many examples of façades drawn by architects in orthographic projection during the design of a building, which indicate a frontal viewing. These kinds of façades can be safely considered a two-dimensional composition with three-dimensional attributes—something comparable to a relief rather than a sculpture in the round. Movement for such objects may help discern the shape, but is not essential to understanding it. Moreover, it would be relatively easy to find a façade that was treated as a stand-alone composition in its own right, over and above how it contributed to the visual design of the entire building. The choice of the façade as a case thus simplified many issues for us without compromising the basic idea to be tested.

**Figure 1:**
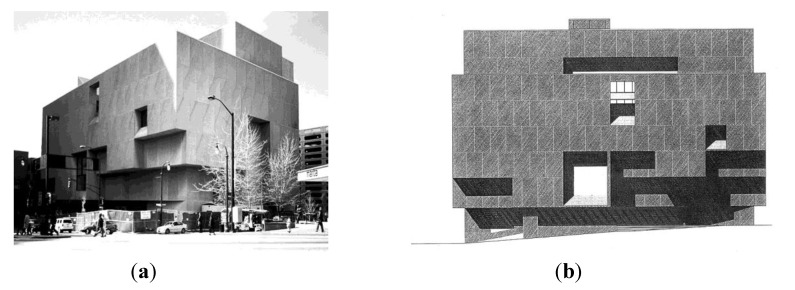
(**a**) Photograph of Atlanta Public Library, Georgia; (**b**) Orthographic elevation drawing of Atlanta Public Library.

The façade selected is the entrance front of the Atlanta Public Library, constructed in 1981 (Figure 1). The architect in charge of construction was Hamilton Smith of Marcel Breuer Architects (MBA), however much of the basic façade design was actually produced under the direction of Marcel Breuer prior to his retirement from the firm in 1974. The façade is a dull gray, monochrome composition of pre-cast concrete panels and glass, which is both severe and uncompromising in its Brutalist aesthetic. Simultaneously, the façade is a very sophisticated composition, providing an unexpectedly apt illustration of a visual composition designed not just to please the eye, but also to engage the mind [17].

Breuer was trained at the Bauhaus under a very particular design philosophy. The core element of design was to create abstract visual compositions that would deliberately bring into play the complex and automatic aspects of human vision that the gestalt psychologists of the time had begun to describe. Such visual forms, these designers believed, would help contemporary man overcome the fragmented and dissociative experiences that the modern world had created and “reform [him] into an integrated being” [18]. To achieve a “dynamic integration”, visual forms had to meet certain criteria: they would have to be plastic (*i.e.*, produce a unified and balanced experience). The characteristics of such dynamic forms included (1) asymmetric; yet (2) balanced composition, which could be resolved into (3) a small set of elementary figures against a ground, but in such a way that (4) the consistency of one reading would be constantly challenged by another; and (5) the identification of the figures against a ground and their inter-relationship would be consistently open to re-evaluation. The relationships between these emergent figures could be described in standard compositional terms—repetition, inversion, symmetry, rhythm, contrast, opposition—the terms demonstrating the various ways in which the viewer might be led to a kind of predictive understanding of the logic of composition. Kepes and others argued that such dynamism was essential for the maintenance of the observer’s attention [18].

The stated design intent to create a composition of dynamic balance, while deploying plastic means, meant that two distinct criteria of performance could be identified for the design of the façade, and each could be associated with a distinct pattern of attention. The first criterion was that the quality of plastic experience produced by the façade. The term plastic, as used by Breuer and his contemporary, characterized a visual experience in which the viewer is able to parse a given object or composition into a set of emergent shapes, to group them in various hierarchies, and to infer various simultaneous relationships between both the individual shapes and their groups. Our normal visual experience has elements of plasticity, but signs of highly developed plasticity are particularly noticeable if one finds attention paid to complex and layered qualities of depth in a dominant two dimensional composition. In the context of the Atlanta library façade, then, a successful plastic experience would be revealed if one found attention given to those elements that showed evidence of compositional relations in depth.

The second intended criterion that we could attribute to the design of the Atlanta library façade was overall dynamism. The dynamism in question here was that of the viewer’s visual activity. A measure of the compositional quality of the façade, therefore, was its ability to distribute the observer’s visual attention evenly over the entire façade rather than focusing more strongly on a local area. Evidence that viewers picked up or noticed peripheral elements as frequently as central ones would make a strong case that the façade composition maintained an overall dynamism.

**Figure 2:**
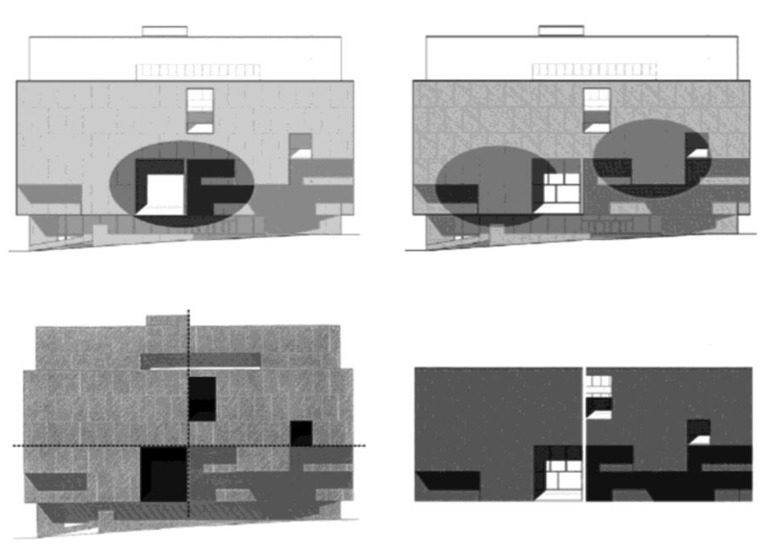
Original façade design—highlighting characteristics of dynamic forms.

The Atlanta Public Library façade, therefore, had several features as a composition that made it particularly well suited for our study. First, it is an abstract composition that can be decomposed easily and relatively unambiguously into a set of discrete elements. This, and the fact that it was a monochrome composition, further reduce the complexity of compositional variables. Second, its design philosophy, as we have seen above, was clear, explicit, and well articulated, thus allowing us to infer intent and to relate it to outcomes of attention that were observable in principle. Finally, the minimalism of the façade composition made it possible for us to design reasonable alternatives (Figure 1 and Figure 2).

All this meant that our general hypothesis could be given a more precise and statistically testable formulation—we could present subjects with two different façade compositions, one altered from the original other, by changes to specific elements that maintained relations like symmetry and repetition, but, in our judgment, impaired the two qualities that Breuer had intended—dynamism and plastic experience. We could note the consequence of these changes by asking the subjects to reproduce these façades under different conditions, and checking which elements or relations they reproduced accurately. Our hypothesis was that even though the changes we made to the façade left most of it intact and maintained its visual language as well as its overall logic, there would be a significant difference in what viewers picked-up or favored when reproducing it. Not just that, we also hypothesized that the differences would show that the altered façade had lost some of its dynamic, plastic experience—that viewers of the altered façade would tend to distribute attention less evenly across it and show less awareness of its depth. In other words, the altered façade was supposed to not perform as well compositionally as the original one, but in ways that are not easily described in language that is conventionally used to describe compositions.

We discuss all this in more detail in the following section.

## 2. Method

### 2.1. Procedure

**Figure 3:**
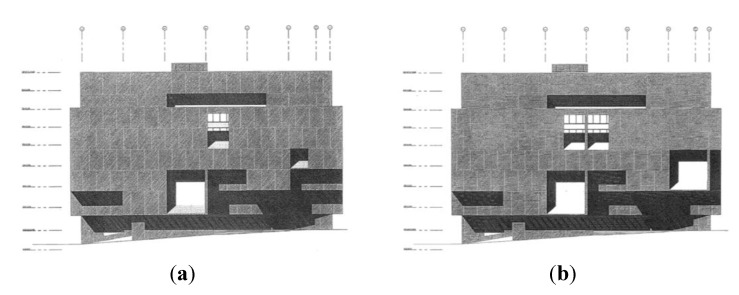
Orthographic Atlanta Public Library façade drawings used for the experiment. (**a**) Design by Marcel Breuer; (**b**) Design composition altered by research team.

Two groups of 12 subjects were given two successive tasks. The subjects were shown a façade in several images, first presented successively, then simultaneously, and then were asked to take their time and memorize a frontal view of it (Figure 3). Following this, they were given two successive tasks, the first to draw from memory the learned view and the second to copy it rapidly from a projected view in a very limited amount of time. The short time was expected to force subjects to prioritize and select features to include—without judgment or time for reflection—which could give clues to their mental organization of the façade. The copy exercise came second since the act of drawing through copying allows processing into memory to occur and would create a bias of familiarity, which would affect the recall study results. Subjects drew these features on a drawing similar to the orthographic drawings shown below with front façade removed. The first set of subjects worked on the façade of Marcel Breuer’s Atlanta Public Library and the second set of subjects on an altered version of the façade. Subjects are compared by subject groups exposed to the original design or altered design and the goal of the study is to compare by groups of subjects not individual subjects, which allow us to capture differences in the object properties of the compositions rather than differences due to personal interpretations.

The first task, a memory exercise, was chosen to help us retrieve the saliences of the façade, which caught the viewing subject’s attention as he or she tried to memorize it. The inferential, attention driven process also guides what is encoded in short term memory and what is remembered; we are selective not just about what we see in our visual environment, but also about what is entered into our long-term memory [13,19]. This implies that if we recall something that was seen, we must have been attending to it when we saw it.

The façade was designed to exhibit a very specific visual organization, which relied on standard compositional moves (Figure 2). The second task, an exercise in copying, is paired with the memory exercise to give us clues to how the eye selectively parses the composition according to its sets of elemental relationships. In order to copy the façade under a time pressure, the subject would have to organize what was in front of him and prioritize what to draw.

A major problem that we faced in designing the experiment was that the same subject could not be asked to work on both versions of the façades—clearly both recall and copying for the second façade would be influenced by exposure to the first in ways that could not be easily listed or discounted. We had, therefore, to assign the façades to different groups. This naturally raised concerns of external validity, and we tried to address these by careful selection of the subjects.

All subjects were drawn from a single cohort of undergraduate architecture students in their third year of architecture design education. All subjects were between 20 and 24 years of age with gender difference balanced. The goal was to select subjects who have a generalized knowledge of architecture—who are trained to look at and produce architectural drawings. Students in their third year are competent enough to handle architectural representation but have very limited exposure to this style of architecture, which prevents a bias of style preference or familiarity with the architect and/or building to occur. The group was equally balanced in terms of academic and design performance, as well as by sex. Although not ideal, we felt that the careful balancing of the subject groups would go a long way in addressing concerns whether our results could be ascribed to differences between facades or between groups. Two additional features of our experimental design would, we thought, further allay these concerns. First, our hypothesis concerned both overall and specific results—while it may be indeed difficult to decide if an overall difference in performance of the two groups was better ascribed to façades rather than to personal differences, the specific results on differences of dynamism and plasticity could only be explained by differences in façades: if results for one façade turned out to show more dynamism or more plasticity, as we hoped, it would be odd to ascribe those results to differences between groups. Second, because we gave the groups two tasks, if we saw a similar difference in performance across both tasks, it would certainly raise serious concerns of performative differences between the two groups. However, if we did not (as turned out to be the case, in the end), this would give us an additional support for believing that the differences were due to façades rather than groups.

The building was presented to subjects through the viewing of multiple images of the building façade successively and then simultaneously. Subjects were limited to viewing only the façade seen through viewing pictures and not in a real-life setting.

### 2.2. The Design of the Alternative Façade and Coding Scheme

It will be recalled that the front façade of the Atlanta public library was chosen for its ability to be decomposed into a set of discrete elements. The coding listed not only basic elements, and some basic emergent elements, but also secondary elements like shadows and supporting graphic elements like the panel-grid that subjects sometimes used to locate the elements. It should be noted that there was no *a-priori* standard of correctness imposed upon the elements. For instance, a number of subjects tended to interpret some of the shadows as figural features of the façade, and we included such shadows in our list of elements characterizing the façade. As our results will show, such emergent but actually false elements could be important data indicative of the type of reading induced by the façades.

**Figure 4:**
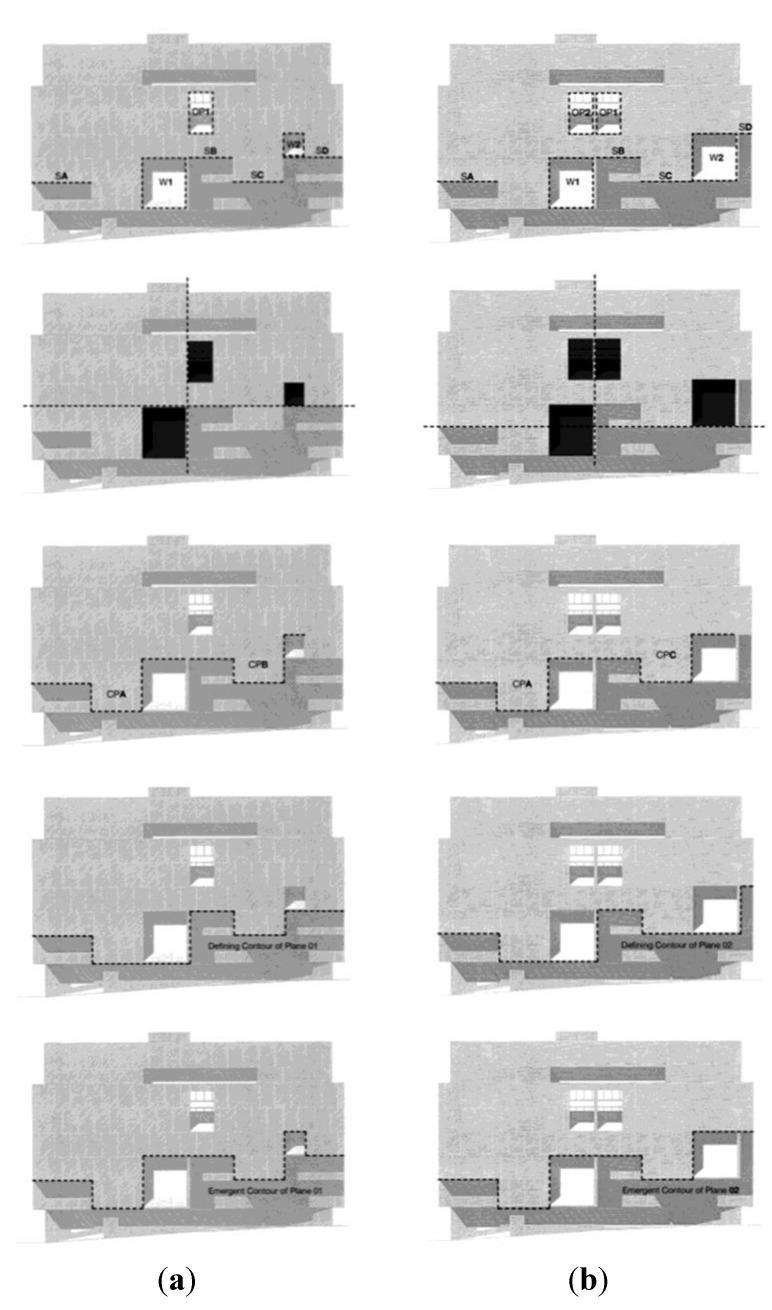
Façade design comparisons and coding. (**a**) Original Design; (**b**) Altered Design.

In the list of elements produced for analysis, most elements remained the same in the two compositions, and where the compositions differed, the differences could be mapped to corresponding elements. This meant that matched pair *t*-tests could be run to compare differences between the elements recorded for the original façades and those for the altered one. Figure 4 clarifies the hierarchy between elements and contour lines, bringing forth simple and emergent overlapping figures and relationships.

We classified and discretized elements into categories that contribute to two overall experiences: (1) Plastic Experience: surface, depth, ambiguous; (2) Dynamism—attentional focus (2D): central, peripheral, and distributed elements.

In the original façade design by Marcel Breuer (Figure 4), the façade is characterized by a composition of floating uninterrupted planes with undulating edges that wrap the library’s rectangular form. These planes appear “punched out” by a lyrical balance of apertures. There is an underlying Line of Vertical Symmetry present that runs vertically along the façade’s outer plane between Window 1 (W1) and Opening 1 (OP1). As the front face/plane of the façade is pulled upward like a skirt, more planes appear and step back, creating a “stepped” sequencing of planes creating the entry space to the library. Step A (SA), Step B (SB), Step C (SC), and Step D (SD) create gentle alignments with one another. SA aligns along an emergent horizontal line with SC. Similarly; a second emergent horizontal line relates SB and SD. The two pairs create a rhythm as they undulate past one another creating new alignments with the three apertures: W1, Window 2 (W2), and OP1. The edge boundaries of W1, SB, W2, and SD create an emergent horizontal “middle”.

Patterning emerges as contours form with bounding edge relationships nested against the two windows. The two primary contour profiles—Contour Profile A (CPA) and Contour Profile B (CPB)—are defined by the edges of the steps and the new edges that W1 and W2 create. The contour profile line is an emerging line made from segment lines at the façade’s edge creating two contour profile lines and a repeating relationship. Relationship 1 (R1) is defined by CPA and W1, and Relationship 2 (R2) is defined by CPB and W2. As noted in Figure 4, the contour profile lines follow the lines of the steps and then snake around the left and top edge lines of the windows. All segment lines to the contour profile lines of CPA repeat identically with CPB except for the top edge line length of W2. This underlying repetition of edge lines with a corresponding element and creates a subtle complexity to visually understanding the organization of the façade.

The façade front plane contains two contour profile edges simultaneously. The first, Defining Contour of Plane 01, is the enveloping edge boundary. The Emergent Contour of Plane 01 is a secondary contour edge that is an emergent figure, lifting the Defining Contour of Plan 01 up around the edges of window W1 and window W2.

As noted earlier, the aim of altering the façade composition was to exaggerate figural relationships, such that it might affect both the perceived dynamism and the plastic experience. In the Altered Drawing relationship, R2 is replaced with relationship R1. This alteration creates a new identical repetition of relationships between relationship R3 and relationship R4. In order to repeat this relationship, window W2 grew in size to mimic window W1 and the bottom edge was dropped to align with step SC. Step SD was brought up to align with the top edge of window W2. While increasing the repetition of elements, a strong sense of symmetry emerged. To increase the symmetry further, a second opening, OP2, was added straddling the Vertical Line of Symmetry. These alterations in compositional elements alter the overall composition so that the plastic experience is lessened and overall dynamism is decreased. These discrete elements can be classed into categories (surface, depth, ambiguous) that contribute to plastic experience, and those that contribute to overall dynamism (central, peripheral, and distributed elements.

Given the increase of overall order through symmetry and repetition in the altered facade, we expected its design to be easier to recall, particularly in relation to the copying exercise. However, we also expected that the less sophisticated design of the altered façade would reflect in a less dynamic distribution of attention and that this effect would be more pronounced during the copying exercise. In effect, the relative compositional complexity of the original façade would make it more difficult for subjects copying details to resolve the façade into a simple hierarchy and errors in reproducing would be more distributed.

## 3. Results

The results supported our general hypotheses. A matched-pair *t*-test showed that subjects tended to recall elements of the altered drawing significantly better than they did corresponding elements of the original (*t* (32) = 3.669, *p* = 0.0009). The matched-pair *t*-test comparing the subjects’ performance during the copying exercise showed the opposite result—subjects tested on the original façade reported marginally greater number of elements than those tested on the altered façade. However, the difference did not reach statistical significance (*t*(32) = −0.956, *p* = 0.34), and we cannot discount that the result was not entirely a matter of chance (Table 1).

**Table 1:** Matched Pairs Comparison: (**a**) Memory Exercise: Overall recalls in Original *versus* Altered Drawings; (**b**) Copy Exercise: Overall reproductions in Original *versus* Altered Drawings.

Memory Exercise—Overall Recalls:			Copy Exercise—Overall Reproductions:	
Recall_Alt	0.61		Reprod_Alt	0.57
Recall_Orig	0.47		Reprod_Orig	0.6
Mean Difference	0.14		Mean Difference	−0.04
Std Error	0.04		Std Error	0.04
Upper 95%	0.21		Upper 95%	0.04
Lower 95%	0.06		Lower 95%	−0.11
N	33		N	33
t-Ratio	3.67694		t-Ratio	−0.965591
DF	32		DF	32
Prob > |*t*|	0.0009		Prob > |*t*|	0.3463
(**a**)			(**b**)	

In other words, alterations made to the façade did allow subjects to remember it better than the original one, but this advantage did not translate to the copying exercise.

**Figure 5:**
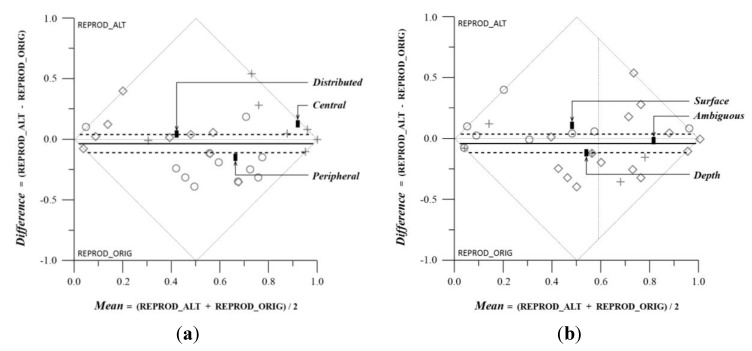
Matched Pairs Comparison on Copy Exercise: Reproductions in Original *versus* Altered Drawings. (**a**) Grouped by Attentional Focus; (**b**) Grouped by Plastic Values.

**Figure 6:**
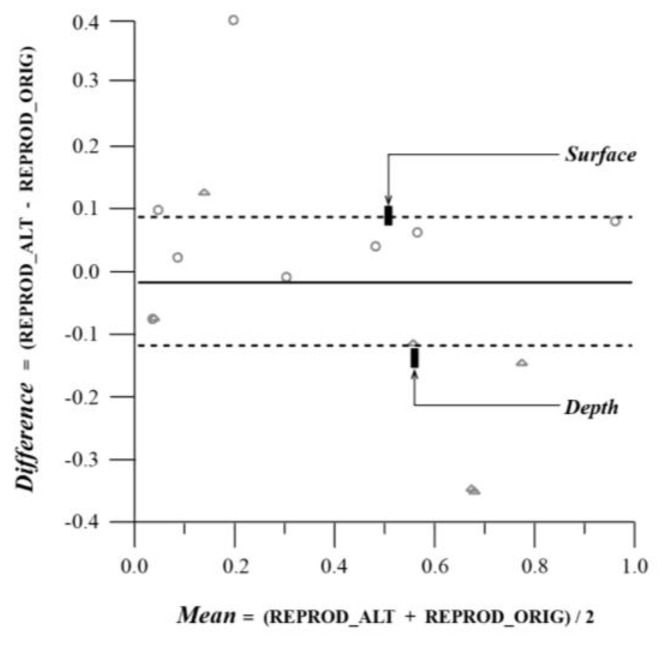
(Alt.) Matched Pairs Comparison on Copy Exercise: Reproductions in Original* versus* Altered Drawings grouped by Plastic Values (Ambiguous Excluded).

Finer analysis of the data revealed more interesting findings regarding our subsidiary hypotheses. We had expected the altered composition to lose some of the original’s plastic quality—in terms of our data, this would imply that the subjects given the original façade would report elements related to depth perception in greater numbers, and they would report elements on the periphery of the façade in greater numbers, indicating a more varied and multi-focal attentional span. This turned out to be partially validated by the results obtained in the copying exercise. Grouping the data by elements recording attentional focus, and running a matched pair analysis, we found that peripheral elements were recorded in greater numbers for the original design, and the central ones for the altered one, and the differences between them were significant (Table 2a and Figure 5a). Grouping elements by plastic values, the differences in either the surface or the depth elements between the altered and original drawings were not significant, though the expected bias towards the depth elements in the original drawing did show up (Table 2b and Figure 5b). However, ambiguous elements were recorded in significantly greater numbers overall, in both drawings, as compared to the surface and depth elements (Across group comparisons table for Plastic Values, Mean Mean column, Table 2a and Figure 5a). When we excluded these ambiguously plastic elements from the data, and ran the matched pair analysis on elements grouped only into surface and depth qualities, we found that the differences between the mean differences recorded for the depth and surface elements was both significant and in the right direction—subjects recalled elements with depth cues in greater numbers in the original design as compared to the altered one (Table 3, Table 4, and Figure 6).

**Table 2:** Matched Pairs Comparison on Copy Exercise: Reproductions in Original *versus* Altered Drawings. (**a**) Grouped by Attentional Focus; (**b**) Grouped by Plastic Values.

*Attentional Focus—Across Groups*					*Plastic Values—Across Groups*			
*Attentional Focus*	*Count*	*Mean Difference*	*Mean Mean*		*Plastic Values*	*Count*	*Mean Difference*	*Mean Mean*
Peripheral	12	−0.19	0.57		Surface	9	0.08	0.41
Central	11	0.09	0.84		Depth	6	−0.15	0.48
Distributed	10	0.005	0.32		Ambiguous	18	−0.05	0.71
*F Ratio*		7.29	15.86		*F Ratio*		2.45	4.37
*Prob>F*		0.0026	<0.001		*Prob>F*		0.103	0.0215
*Pairs/Axis*		Within/Y	Among/X		*Pairs/Axis*		Within/Y	Among/X
(**a**)					(**b**)			

**Figure 7:**
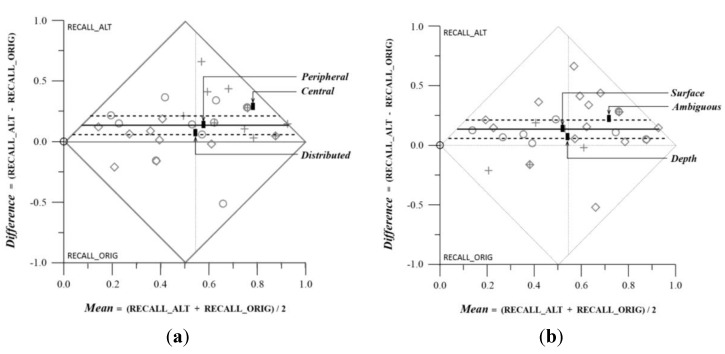
Matched Pairs Comparison on Memory Exercise: Recalls in Original *versus* Altered Drawings. (**a**) Grouped by Attentional Focus; (**b**) Grouped by Plastic Values.

The drawing from memory exercise did not produce significant differences; the mean difference by groups showed that the mean differences between original and altered drawings was lower for the peripheral elements as compared to the surface ones (Table 3, Table 4, and Figure 6) but the difference between the two groups was not significant. Grouping by plastic characteristics produced similar results: the direction of the results was according to our hypotheses, but the results failed to reach an acceptable level of significance (Table 5 and Figure 7).

**Table 3:** (Alt.) Matched Pairs Comparison on Copy Exercise: Reproductions in Original *versus* Altered Drawings grouped by Plastic Values (Ambiguous Excluded).

Copy Exercise—Overall Reproductions with Ambiguous Excluded:	
Reprod_Alt	0.43
Reprod_Orig	0.44
Mean Difference	−0.01
Std Error	0.05
*t*-Ratio	−0.29419
DF	14
Prob > |*t*|	0.7729

**Table 4:** (Alt.) Matched Pairs Comparison on Copy Exercise: Reproductions in Original* versus* Altered Drawings grouped by Plastic Values (Ambiguous Excluded).

Plastic Values—Across Groups			
Plastic Values	Count	Mean Difference	Mean Mean
Surface	9	0.08	0.41
Depth	6	−0.15	0.48
F Ratio		8.22	0.15
Prob>F		0.0132	0.7046
Pairs/Axis		Within/Y	Among/X

Summing up, the altered drawing was easier to recall, but not to reproduce; when reproducing the altered drawings the subjects tended to reproduce elements related to surface composition and those which were centrally located in far greater numbers than the subjects who worked on the original drawings. When recalling the façades, similar differences, although observed, were not reliably large.

**Table 5:** Matched Pairs Comparison on Memory Exercise: Recalls in Original *versus* Altered Drawings. (**a**) Grouped by Attentional Focus; (**b**) Grouped by Plastic Values.

*Attentional Focus—Across Groups*					*Plastic Values—Across Group*			
*Attentional Focus*	*Count*	*Mean Difference*	*Mean Mean*		*Plastic Values*	*Count*	*Mean Difference*	*Mean Mean*
*Peripheral*	12	0.11	0.48		*Surface*	9	0.11	0.45
*Central*	11	0.26	0.71		*Depth*	6	0.04	0.48
*Distributed*	10	0.04	0.44		*Ambiguous*	18	0.19	0.61
*F Ratio*		3	5.24		*F Ratio*		1.24	1.8
*Prob>F*		0.0651	0.0112		*Prob>F*		0.3049	0.1835
*Pairs/Axis*		Within/Y	Among/X		*Pairs/Axis*		Within/Y	Among/X
(**a**)					(**b**)			

## 4. Discussion

Two points need to be noted to further interpret the results. First, our hypotheses worked out largely in the copying exercise only—the results from the recall exercise were ambiguous. Although we had not formulated an explicit hypothesis about this result at the outset, the result does seem to accord with common experience. Qualities like overall plasticity and ability to distribute attention are not aids to better memory, but rather to heightened perceptual richness and the vividness with which a composition like a façade is experienced. So, while looking at the façade the engaged viewer might attend to details that increase plasticity and distribute attention, he or she is not likely to be aware of such details consciously enough to reproduce them from memory. Attention to such details, therefore, has the curious quality of not entering the viewer’s overt consciousness. Such attention might perhaps be the reason why we cannot enjoy a painting or a song as much by recalling as we do while actually looking at or listening to it. There is some literature on such types of attention but it remains speculative [11]; experiments like ours may help provide some data for further work in this direction.

Second, the statistical results aside, the actual drawing data generated by our experiment gave us further insights into the way complex design compositions are parsed and understood. The key insights came particularly in context of the top-down aspects of this process—that is, in the cognitively driven aspects of understanding visual compositions, over and above the more general perceptual, bottom-up aspects of the parsing process. We observed that subjects constructed distinctive schemas as a procedure for organizing the visual data needed to complete the drawing task. Naturally, our discussion below is speculative, but it is still useful in pointing to directions for further work.

### Schemas

In closing, we think it useful to offer a closer look at the data we collected, over and above, what we used to test hypotheses. In particular, we found it interesting to investigate what strategies our subjects used in addressing the tasks they completed. What follows is a speculative account of these strategies—offered more in the spirit of raising questions for further work rather than providing answers.

Both activities that subjects were asked to perform, to memorize and to copy the façade, would require that the façade be represented in some organized or structured form in the mind. This is not only true of recall activity, but also of copying, since the subjects were given a very short time to reproduce it, and would have to figure out some way to prioritize what they copied. Of the different organizational forms that cognitive scientists have discussed, “schema” seems to be most relevant or applicable here.

Thus, we use the word “schema” to describe this framework that has been constructed by the participant in order to cognitively organize the visual data into a new representation for completing the task. Schema, as defined by cognitive psychologist George Mandler, is a “bounded, distinct, and unitary representation” and a method of “organizing experience” [20]. The level of schema development is dependent upon the level of attention paid to the details of the building and the ability to recognize surfaces and relationships between edges and figures. Two types of schemas were identified.

In the first schema, the Organizational Schema, the organization of elements and relationships between contours and elements are parsed into groups. First subjects recognized the white rectangle as a surface, second, they carved away the contours of the surface in simple rectangular geometries, and lastly they placed architectural elements in the form of simple rectilinear objects in the surface. The second step of the organizational schema describes the way in which participants created the contours of the surface. Contours were created either by carving away the edges of the surface through re-describing them as simple squares or multiple rectilinear geometries that created a more complex contour profile. The advanced organizational schemas show that more attention is paid to the shape complexity of the contour profile. The carved corners of the less advanced schema are rectangular chunked figures in all cases. Elements are drawn as simple figures and are placed with no relationship to the edges of the contour line. In the advanced instantiations of the organizational schema, participants synthesize the anchoring relationships between elements and contour lines. Here, the contour edge of the surface is sculpted away, showing attention paid to the contour line as the undulating boundary edge of the surface, in effect creating the Defining Contour Profile. Elements are placed along this contour line, highlighting the relationship between the contour and the element. The contour profile edges and Vertical Line of symmetry are used as anchors to locate the elements.

In the second schema, the Surface Schema, patterns and underlying marks are drawn as a way to divide the surface—an approach to the parsing of the surface into zones. Students use construction lines in creating surfaces, parsing zones, and aligning elements. Several light primary markings are seen underneath heavier strokes, indicating that the participants use the elevation and column line markers to emulate the texture of the surface, align contour lines, and place elements along the surface edges and the line of symmetry.

## 5. Conclusions

The statistical results from our exercise were largely consistent with our basic hypothesis that the façade designed by Marcel Breuer has both a plastic quality and overall dynamism, and that these qualities depend upon quite subtle design moves. The value of these results is to help confirm the designer’s intuition that simple rule-following moves such as creating local symmetry or repetition do not by themselves lead to successful composition.

The alternative version of the façade we produced was not intended to be better or worse than the original design, as typical explanations of what constitutes better design does not hold, but was simply intended to open up discussion as to why the original design by Breuer was so good. During the design process, the architect is constantly entertaining alternate compositional relationships of building elements throughout the design phase at both a macro (building form) and micro (building details) level. Often increasing order through composition of elements alone is considered a method to increase the quality of architectural designs. This study supports the position that it is not just pure formal qualities, but the distribution of attention, playing a part in the evaluation of a better composition, a better façade design. By distribution of attention, we mean that the designs evaluated as “better” or “good” will tend to distribute attention across more parts of the whole composition through the compositional play of elements that create ambiguities between surface and depth.

A limitation built into this study is that these findings are possibly more relative to people with a specialized background in architecture. Since this is a preliminary study, the specific rules cannot yet be generalized to other aspects of a building design, such as other architectural styles or aspects concerning overall building shape or the generation of form through massing. However, the schemas employed in this experiment demonstrate a top-down processing influenced by the compositional organization of the architectural design. By correlating schema type to architectural composition rules for design, we can begin to establish certain general principles by which the role of the imaginative function shapes understanding of building design. Thus, the importance of this study lies in producing some evidence, which confirms that alterations in forms can result in differences in distribution of attention. This is a contribution to architectural theory and the first step towards building up a theory. We intend to further explore developing systematic procedures for describing architectural design.
